# Supplementary figures and images for: Disorder strength measured by quantitative phase imaging as intrinsic cancer marker in fixed tissue biopsies
Source: PLoS One. 2018 Mar 21;13(3):e0194320. doi: 10.1371/journal.pone.0194320 (PMC5862460; doi:10.1371/journal.pone.0194320)

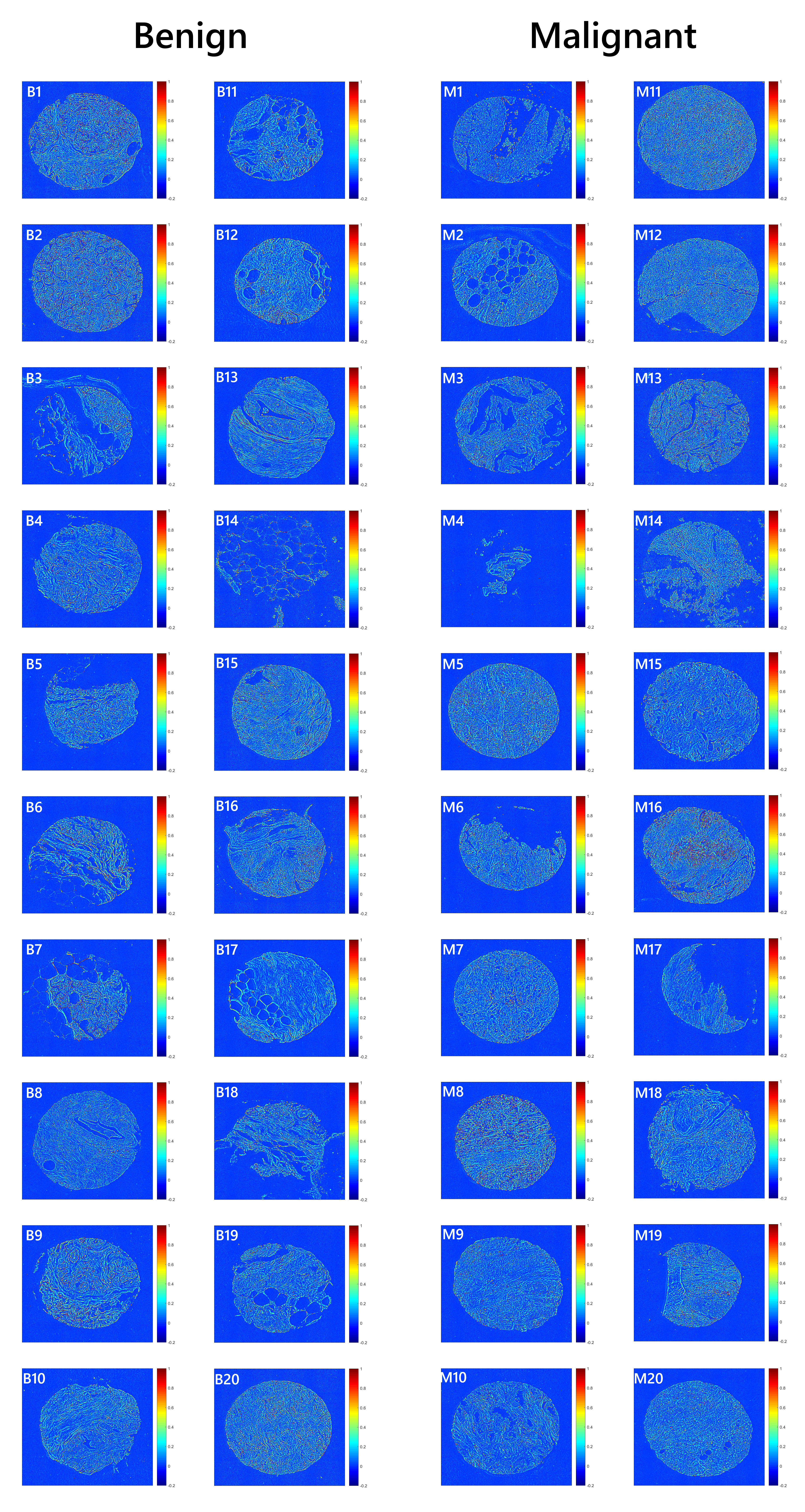

Supplement: S1 Fig — (TIF) [file pone.0194320.s001.tif]

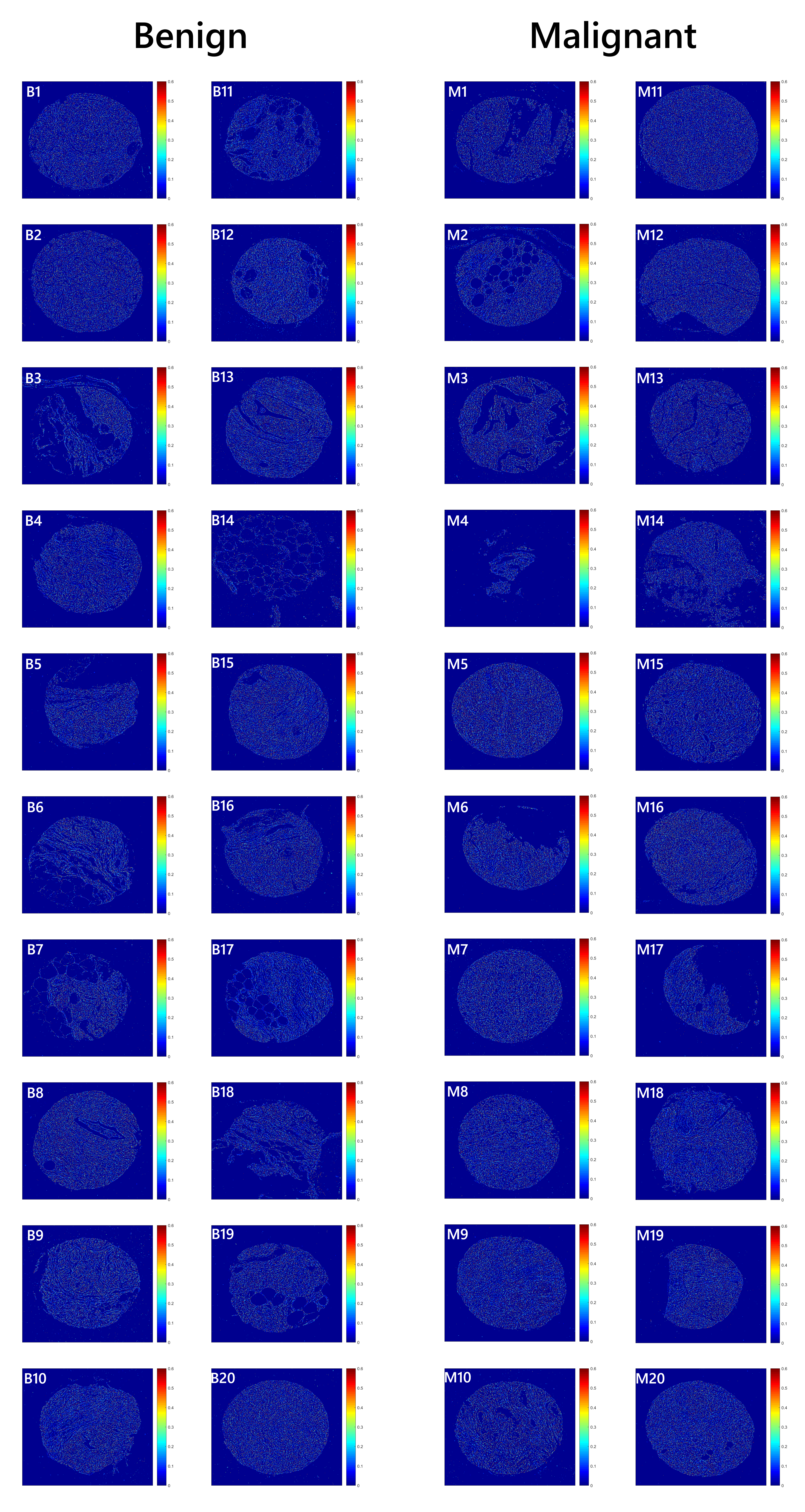

Supplement: S2 Fig — (TIF) [file pone.0194320.s002.tif]
